# Single-cell transcriptomes reveal heterogeneity of chlorine-induced mice acute lung injury and the inhibitory effect of pentoxifylline on ferroptosis

**DOI:** 10.1038/s41598-023-32093-7

**Published:** 2023-04-26

**Authors:** Chen-qian Zhao, Chong Wang, Meng-meng Liu, Meng Cao, Jie Peng, De-qin Kong, Xiao-ting Ren, Rui Liu, Chun-xu Hai, Xiao-di Zhang

**Affiliations:** 1 Department of Medical Experiment Center, Shaanxi University of Chinese Medicine, Xi’an, 712046 Xianyang China; 2 Department of Toxicology, Shaanxi Key Lab of Free Radical Biology and Medicine, The Ministry of Education Key Lab of Hazard Assessment and Control in Special Operational Environment, School of Public Health, Air Force Medical University, Xi’an, 710032 China; 3 Department of Health Service, Logistics University of Chinese People’s Armed Police Force, Tianjin, 300309 China

**Keywords:** Diseases, Health care, Medical research

## Abstract

To investigate the effect of pentoxifylline (PTX) on Chlorine (Cl_2_)-induced acute lung injury (ALI) by single-cell RNA sequencing (scRNA-seq). Female BALB/c mice were exposed to Cl_2_ at 400 ppm for 15 min. H&E staining was used to observe the degree of lung injury. scRNA-seq was conducted to analysis of normal and Cl_2_-exposed mice lung tissues. Immunofluorescence was used to observe genes of interest. Thirty-two mice were randomly divided into four groups: Control, Cl_2_, Cl_2_+Fer-1, Cl_2_+PTX. TEM, WB and ELISA were used to detect ferroptosis-related indicators. The 5, 8, 10, 12, 16, 20 clusters were epithelial cells and 4, 15, 18, 19, 21 clusters were endothelial cells. Pseudo-time analysis revealed the differentiation trajectory of epithelial cells and key regulatory genes (Gclc, Bpifa1, Dnah5 and Dnah9) during the process of injury. Cell–cell communication analysis identified several important receptor–ligand complexes (Nrp1-Vegfa, Nrp2-Vegfa, Flt1-Vegfa and Flt4-Vegfa). Ferroptosis were found up-regulated in epithelial and endothelial cells by GSVA analysis. Highly expressed genes to which closely related ferroptosis were found by SCENIC analysis. PTX could significantly decrease the levels of MDA and abnormal high expression of solute carrier family 7 member 11 (SLC7A11, the key transporter of cystine) as well as increase the expression of GSH/GSSG and glutathione peroxidase 4 (GPX4) (*p* < 0.05). This study revealed novel molecular features of Cl_2_-induced ALI. PTX may be a potential specific drug by inhibiting the process of ferroptosis in epithelial and endothelial cells.

## Introduction

Chlorine (Cl_2_), as one of the most widely produced chemicals around the world, is an asphyxiating toxicant. Because of its easy production, Cl_2_ was used on a large scale by the German army in World War I. Inhalation of Cl_2_ can cause cough, shortness of breath and acute lung injury (ALI), permeable pulmonary edema, acute respiratory distress syndrome (ARDS) and even death^1,2^. Numerous studies have reported that the development of ALI is associated with disruption of the alveolar epithelial barrier and increases capillary endothelial permeability^3–5^. As two main components of the blood air barrier, the early molecular events of the epithelial and endothelial cells remain unclear after Cl_2_-exposure, which limits the research and development of effective medicine.

Pentoxifylline (PTX) can improve vascular microcirculation through reduction the plasma viscosity, and increase in erythrocyte flexibility and inhibition the activation of neutrophils^6^. The generally believed molecular mechanism behind these effects is that PTX, as a non-specific phosphodiesterase inhibitor, leads to the increase of intracellular cyclic adenosine monophosphate (cAMP) concentration^7,8^. Interestingly, PTX has also been used in the treatment of COVID-19 but merely in clinical observation^9,10^. The deeper mechanism of PTX which improves ALI needs further exploration and research. We hypothesized that PTX had a good interventional effect by protecting epithelial and endothelial cells after Cl_2_-exposed.

Recently, different from traditional sequencing mixed sample, single-cell RNA sequencing (scRNA-seq) technology has been developed and has revealed characterization of the heterogeneity between cells. In this study, we analyzed the transcriptomic profiles of 5316–7742 cells from normal and Cl_2_-exposed samples. This study observed the single-cell expression profiles of epithelial and endothelial cells lineages as well as intercellular communication between them, tried to establish their correlation. Based on the new finding (ferroptosis), the intervention effect of PTX was revealed. We hope to provide experimental basis for the research and development of specific drugs for the treatment of Cl_2_ injury.

## Materials and methods

### Experimental animals

Female BALB/c mice aged 6–8 weeks old were purchased from the animal centre of the Fourth Military Medical University (Xi'an, China) and housed in a controlled environment (22 ± 2 °C, 12 h light/dark cycle, free access to food and water). Before the experiment, the mice were fasted for 12 h. All procedures involving animals and their care were performed in accordance with National Institutes of Health (NIH Pub. No. 85-23, revised 1996), and approved by the Animal Care and Utilization Committee of the Air Force Medical University and our reporting follows the recommendations in the ARRIVE guidelines.

### Cl_2_ exposure and experiment groups

First, 6 mice were randomly assigned into 2 groups: Control and Cl_2_ groups_._ In another experiment, 32 mice were randomly assigned into 4 groups: Control, Cl_2_, Cl_2_+Fer-1 (Ferrostatin-1, ferroptosis inhibitor, 347174-05-4, TargetMol), Cl_2_+PTX (6493-05-6, Sigma). Mice were put in the Cl_2_ exposure chamber in batches. The exposure condition was 400 ppm and mice were exposed to this concentration of Cl_2_ for 15 min. The control mice stayed in the same chamber for 15 min without Cl_2_.

Cl_2_+Fer-1 and Cl_2_+PTX groups were given Fer-1 (5 mg/kg) and PTX (10 mg/kg) by intraperitoneal injection and intragastric administration immediately after Cl_2_ exposure, with a volume of 0.2–0.25 ml per mouse. Fer-1 was diluted with 0.01% DMSO in saline to a final concentration of 0.5 mg/ml.

Then mice were placed in the indoor air and injected intraperitoneally with 2% Pentobarbital Sodium (40 mg/kg) 3 h later. After anesthesia, the animals were fixed on the animal dissection platform, and their lung tissues and blood were taken.

### Preparation of single-cell suspensions

Lung tissues was removed after 3 h from normal and Cl_2_-exposed mice and promptly sent to the research facility. Each sample was subsequently minced on ice to less than 1 mm cubic pieces, followed by enzymatic digestion using Sigma with manual shaking every 5 min. Samples were then centrifuged at 300 rcf for 30 s at room temperature and the supernatant were removed without disturbing the cell pellet. Next, 1 × PBS (calcium and magnesium free) containing 0.04% weight/volume BSA (400 µg/ml) was added and then centrifugated at 300 rcf for 5 min. The cell pellet was resuspended in 1 ml red blood cell lysis buffer and incubated for 10 min at 4 °C. After Red Blood Cell Lysis, samples were resuspended in 1 ml PBS containing 0.04% BSA. Next, samples were filtered over Scienceware Flowmi 40-µm cell strainers (VWR). After lung tissue dissociation, cell concentration and cell viability were determined by hemocytometer and Trypan Blue staining.

The nuclear suspension preparation experiment was conducted on ice. Cut fresh or frozen tissue samples in 1 ml of nuclear lysate (NST). The components of NST are 0.1% NP40, 10 mM Tris–HCl, 146 mM NaCl, 1 mM CaCl_2_, 21 mM MgCl_2_ and 40 U/ml RNase inhibitor. The cracking time is 7 min. After confirming the complete nuclear lysis by trypan blue staining microscopy, add 1 ml ST Wash buffer (10 mM Tris–HCl, 146 mM NaCl, 1 mM CaCl_2_, 21 mM MgCl_2_, 0.01% BSA (NEB B9000S) and 40 U/ml RNase inhibitor). 40 µm cell sieve (BD) is filtered, the filtrate is transferred to a 15 ml centrifuge tube, the cell sieve is washed with an appropriate amount of ST Wash buffer, and the washing solution is combined with the nuclear filtrate. Centrifuge the horizontal rotor at 500 g for 5 min at 4 °C. The nucleus was resuspended with 5 ml PBS + 1% BSA. After washing and centrifugation, 100 µl PBS + 1% BSA resuspended the nucleus. Trypan blue staining, microscopic count.

Use PBS + 1% BSA to dilute the nucleus to a concentration of 700–1200/µl. According to 10 × Genomics Chromium Next GEM Single Cell 3′ Reagent Kits v3.1 (1000268) operating instructions are used for computer operation and cDNA library amplification. DNA library construction adopts Chromium™ single cell 3′/5′ library construction kit (1000020). The constructed library was sequenced on the Illumina Nova 6000 platform using PE150 sequencing mode.

### scRNA-seq

The Seurat R package^11^ was applied to convert scRNA-seq data into Seurat objects in our study (Supplementary information 1). Cells expressing less than 200 genes or more than 7000 genes or with more than 20% mitochondrial genes were removed in the quality control step. The data was then normalized with the NormalizeData function using the LogNormalize method, and FindVariableFeatures was used to identify the top 2000 highly variable genes. Next, we used the RunPCA function to reduce the dimensionality of the scRNA-seq data after scaling and centering the features by using the ScaleData function. The RunHarmony function in the Harmony R package can take into account of multiple factors simultaneously, and can be used with default parameters to integrate the different study cohorts in our study^12^. We then perform a Unified Manifold Approximation and Projection (UMAP) analysis using the RUNUMAP function. We also used the FindClusters and FindAllMarkers functions for cell clustering analysis and detection of gene expression markers^13^.

The SubsetData function is also used to extract subclusters for downstream analysis. After detecting clusters and gene expression markers in subclusters with the FindClusters and FindAllMarkers functions, UMAP analysis was also conducted using the RUNUMAP function. Subclusters are annotated as described above.

### Gene Ontology (GO) and Kyoto Encyclopedia of Genes and Genomes (KEGG)

Differentially expressed genes (DEGs) were identified using the FindMarkers function (test.use = presto) in Seurat. P value < 0.05 and |log2foldchange|> 0.58 was set as the threshold for significantly differential expression. GO enrichment and KEGG pathway enrichment analysis of DEGs were respectively performed using R based on the hypergeometric distribution.

### Genome variation analysis (GSVA)

To conduct the Gene Set Variation Analysis, the GSEABase package (version 1.44.0) was used to load the gene set file which was downloaded and processed from KEGG database (www.kegg.jp/kegg/kegg1.html). To assign pathway activity estimates to individual cells, we applied GSVA using^14^ standard settings, which was implemented in the GSVA package (version 1.30.0, http://www.bioconductor.org/packages/release/bioc/html/GSVA.html). The differences in pathway activities scored per cell were calculated with LIMMA package (version 3.38.3).

### Pseudo-time analysis

We determined the developmental pseudo-time with the Monocle2 package^15^. The raw count was first converted from Seurat object into CellDataSet object with the ImportCDS function in Monocle. We used the differential GeneTest function of the Monocle2 package to select ordering genes (qval < 0.01) which were likely to be informative in the ordering of cells along the pseudo-time trajectory. The dimensional reduction clustering analysis was conducted with the ReduceDimension function, followed by trajectory inference with the OrderCells function using default parameters. Gene expression was plotted with the plot genes in pseudo-time function to track changes over pseudo-time.

### Single-cell regulatory network inference and clustering (SCENIC) analysis

The SCENIC analysis was conducted using the motifs database for RcisTarget and GRNboost (SCENIC^16^ version 1.1.2.2, which corresponds to RcisTarget 1.2.1 and AUCell 1.4.1) with the default parameters. In detail, we identified transcription factor (TF) binding motifs over-represented on a gene list with RcisTarget package. The activity of each group of regulons in each cell was scored by AUCell package.

To evaluate the cell type specificity of each predicted regulon, we calculated the regulon specificity score (RSS), based on the Jensen–Shannon divergence (JSD), a measure of the similarity between two probability distributions. Specifically, we calculated the JSD (Jensen-Shannon divergence) between each vector of binary regulon activity overlaps with the assignment of cells to a specific cell type^17^. The connection specificity index (CSI) for all regulons was calculated with the scFunctions (https://github.com/FloWuenne/scFunctions/) package.

### Cell–cell communication analysis

We used the CellPhoneDB (v2.0) to identify biologically relevant ligand–receptor interactions from scRNAseq data. We defined a ligand or a receptor as “expressed” in a particular cell type if 10% of the cells of that type had non-zero read counts for the ligand/receptor encoding gene. Statistical significance was then assessed by randomly shuffling the cluster labels of all cells and by repeating the above steps a null distribution was generated for each LR pair in each pairwise comparison between two cell types. After running 1000 permutations, P values were calculated with the normal distribution curve generated from the permuted LR pair interaction scores. R packages Igraph and Circlize were used to display the cell–cell communication networks.

### Histology and immunofluorescence

Mice were anesthetized with 2% Pentobarbital Sodium. After opening the chest cavity, each mouse was bled through the abdominal aorta and cold PBS was perfused through the right ventricle to further clear the lungs of blood. Lungs were dehydrated, embedded in paraffin, and sectioned. Morphology was assessed by Hematoxylin–eosin (H&E) staining. Immunofluorescence was used to identify various endothelial and epithelial antigens using the following antibodies: CK18 (GB11232, Servicebio); CD31 (GB113151, Servicebio); FoXO1 (GB11286-1, Servicebio); FoXO3 (66428-1-Ig, Proteintech); GPX4 (ORB135590, Servicebio); SLC7A11 (26864-1-AP, Proteintech); Gclc (12601-1-AP, Proteintech); Baifal (10413-1-AP, Proteintech).

### Transmission electron microscopy examination (TEM)

Lung tissues were obtained immediately after anesthesia of the mice and cut into small pieces (1 mm^3^). The specimens were fixed with 2% glutaraldehyde at 4 °C, washed in the 0.1 mol/l phosphate buffer (pH 7.4), fixed with 1% osmium tetroxide, and stained with 1% aqueous uranyl acetate. After dehydration through an ethanol series, the specimens embedded in Epon 812 and ultrathin sections were collected on copper grids. The specimen sections were stained with uranyl acetate and alkaline lead citrate respectively and observed in TEM of Jeol JEM 1400 (Tokyo, Japan).

### Western blot (WB)

The total protein concentration of different groups was detected using the BCA Protein Assay Kit (Beyotime). Protecin (30 µg) in the total cell lysates from the lung tissue were separated by SDS-PAGE and transferred to PVDF (polyvinylidene fluoride) membranes. WB was performed using the following primary antibodies: SLC7A11(orb100617, biorbyt); GPX4 (orb135590, biorbyt); β-Actin (orb625112, biorbyt).

### Enzyme-linked immunosorbent assay (ELISA)

In the study, pentobarbital was used to anesthetize the mice in each group, with 8 mice in each. Then the blood was collected by the orbital sinus extraction procedure, and the serum was separated for the subsequent experiment. According to the manufacturer's instructions, the concentrations of SLC7A11 (F30961-A, Fankew, Shanghai FANKEL Industrial Co., Ltd) and GPX4 (F9445-A, Fankew, Shanghai FANKEL Industrial Co., Ltd) in the serum were measured by systems ELISA kits.

### Measurements of MDA and GSH/GSSG levels

The level of MDA was determined using a Lipid Peroxidation Assay Kit (A003-1, Nanjing Jiancheng Bioengineering Institute, Nanjing, China).

The level of GSH/GSSG was determined using GSH/GSSG Ratio Detection Assay Kit (A061-1, Nanjing Jiancheng Bioengineering Institute, Nanjing, China).

### Statistical analysis

Data were expressed as the mean ± SEM. Differences were analyzed using an unpaired two-sided Student’s t test for a two-group comparison or one-way ANOVA test followed by a Bonferroni post hoc test for multiple comparisons. GraphPad Prism statistics was used for the analysis. P < 0.05 was considered statistically significant.

### Ethics approval and consent to participate

The experiment protocols were approved by the local animal ethics committee. All applicable international, national, and/or institutional guidelines for the care and use of animals.

## Results

### A single-cell atlas of the normal and Cl_2_-exposed mice

**Figure 1:**
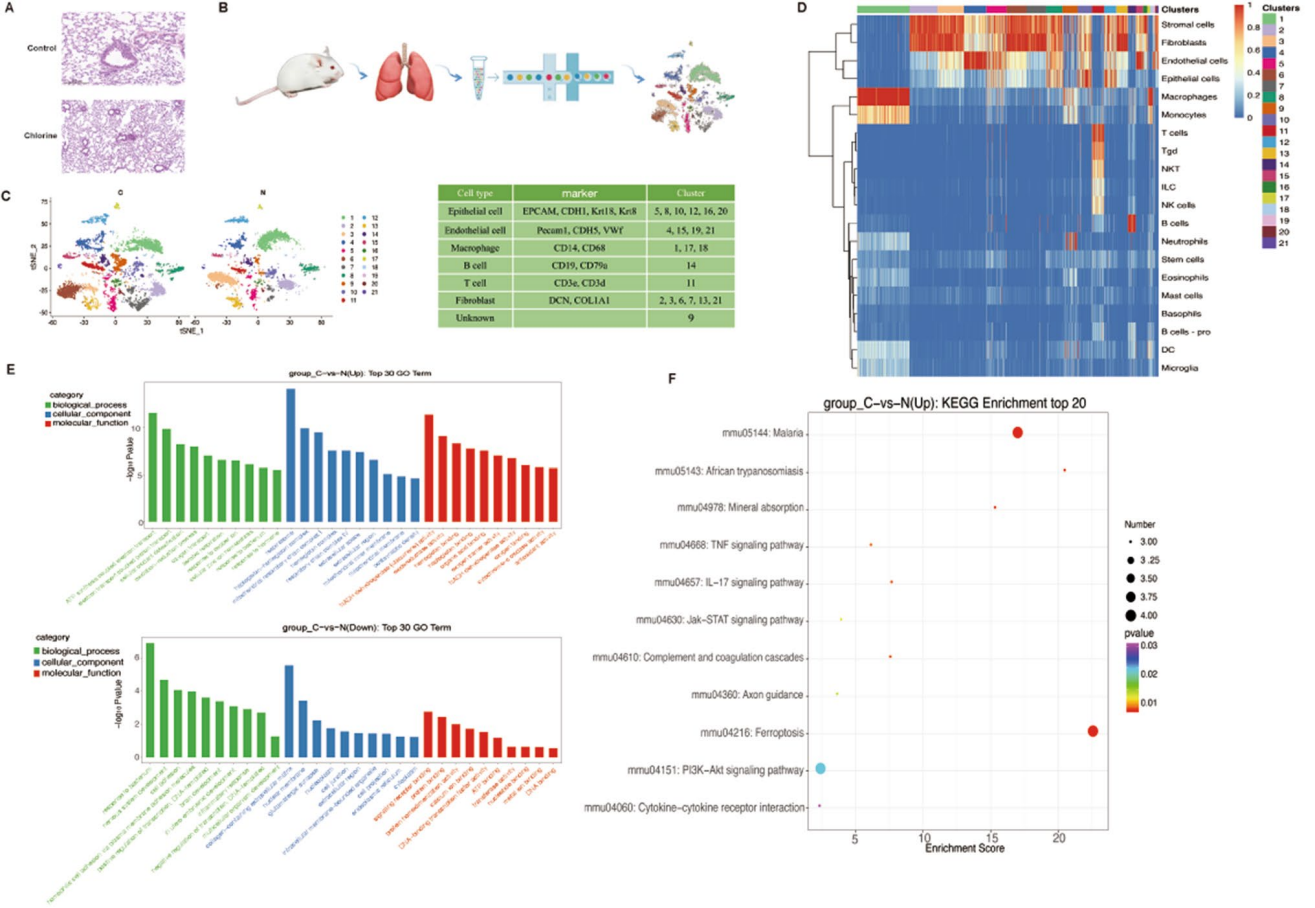
Single-cell transcriptomic atlas of mice lung tissues. (**A**) Lung histology 3 h after exposure to 400 ppm Cl_2_ for 15 min, n = 6. (**B**) Flowchart of experiments in this study. (**C**) Unsupervised clustering of mice lung tissues revealed 21 distinct clusters. Cell name, marker genes and cluster of each cell type were summarized in the right panel. Raw data of cell types are shown in Supplementary information 2. (**D**) The heat map for identification of cell types. (**E**) The GO analysis of top 30 up-regulation and down-regulation in control and Cl_2_ exposure groups. (**F**) The KEGG analysis of top 20 in control and Cl_2_ exposure groups. *N* control group, *C* Cl_2_ group.

After Cl_2_ exposure, lung consolidation occurred in the lung tissues. Moreover, there are small quantity of red blood cells emerging in alveolar spaces (Fig. 1A).

Through the scRNA-seq data analysis, 5316–7742 cells were filtered from normal and Cl_2_-exposed mice including mass postfiltering, of which 9659 cells originated from Cl_2_-exposure and 6226 from normal lung tissues (Fig. 1B). As shown in Fig. 1, the cells were divided into 21 groups by PCA and UMAP clustering methods. By analyzing variably expressed genes across all cells, we identified six major cell types (Fig. 1C). For a more visually presentation of the cell types in each cluster, we provide a heat map of the cell types (Fig. 1D). This illustrates the heterogeneity between different cell types. To understand differences in two groups at a functional level as well as in an expressed signaling pathway, we further characterized gene expression differences between control and exposure groups. By using Gene Ontology (GO) (Fig. 1E), we found that compared with the control group, the ATP synthesis coupled electron transport, electron transport coupled proton transport, cellular oxidant detoxification, oxidation–reduction process, oxygen transport, aerobic respiration and other functions in the exposure group were up-regulated, while the biological functions such as response to bacterium, nervous system development, cell adhesion, homophilic cell adhesion via plasma membrane adhesion molecule, positive regulation of transcription, DNA-templated, brain development were down-regulated. Kyoto gene and genome (KEGG) enrichment analysis found ferroptosis and PI3K/AKT signaling pathway were highly expressed in the up-regulated signaling pathway (Fig. 1F).

### Pulmonary epithelial cells heterogeneity in the mice

**Figure 2:**
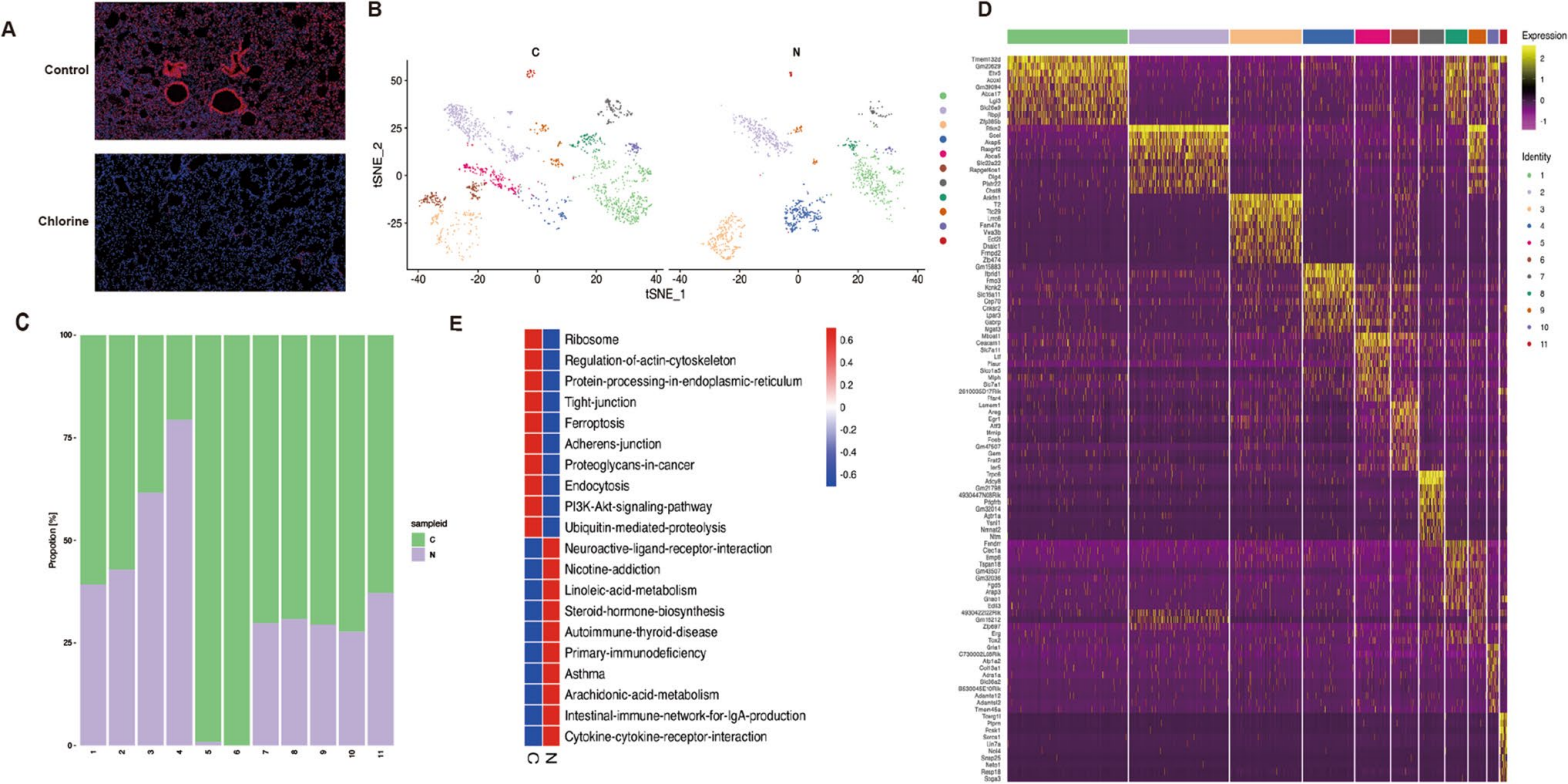
The single-cell transcriptomes of epithelial cells. (**A**) Immunofluorescence staining of epithelial cell CK8 gene in normal (n = 6)/Cl_2_-exposed (n = 6) lung tissues, scale bars 50 μm, CK8 (red), cell nuclear (blue). (**B**) The t-SNE plot and overview of the epithelial cells. (**C**) Proportions of normal and Cl_2_ injury epithelial cells in each subgroups. Raw data of proportions of epithelial cells are shown in Supplementary information 3. (**D**) The heatmap shows the top 10 SDE genes in each subgroups of epithelial cells. (**E**) GSVA analysis indicated enrichment pathways of each subgroups of epithelial cells. *N* control group, *C* Cl_2_ group.

We conducted immunofluorescence staining of normal and Cl_2_-exposed mice lung tissues using CK8, a specific marker gene for epithelial cells. The results suggested that lung epithelial cells were damaged after Cl_2_-exposed (Fig. 2A). At the same time, to depict their intrinsic portraits, these epithelial cells were further divided into eleven subclusters (epithelial cells cluster 1 to 11) by the SNN algorithm and t-SNE analysis (Fig. 2B). Interestingly, clusters 5 and 6 emerged after Cl_2_ exposure (Fig. 2B). Compared with the control group, the subclusters of the lung epithelial cells in the Cl_2_ exposure group were significantly changed: the proportion of clusters 5, 6, 7, 8, 9, 10, 11 were increased, and only the proportion of cluster 4 was decreased (Fig. 2C). The unique gene characteristics associated with each epithelial subgroup and the top 10 SDE genes were described (Fig. 2D). Next, to evaluate which biological functions were involved in the Cl_2_ injury, we conducted GSVA analysis in normal and Cl_2_-exposed mice lung tissues (Fig. 2E). Cluster1 with highly expressed SLC26A9 is a newly discovered member of the multifunctional anion transport family, which is mainly expressed in the mucosal epithelial cells of the lung and stomach (Fig. 2D). From Fig. 2E, we found that the regulation of the cytoskeleton was highly expressed in the Cl_2_-exposed group compared with the control group, and its related genes were distributed in multiple subgroups. For example the gene Tmem132d was expressed in cluster 1, Dlg4 in cluster 2, and Cnksr2 in cluster 4 and Fgd5 in cluster 9 (Fig. 2D). Compared with the control group, ferroptosis and PI3K/AKT signaling pathways were highly expressed in Cl_2_-exposed group (Fig. 2E). Ferroptosis was highly expressed in Cl_2_-induced ALI verified by SLC7A11, a high expression gene of cluster 5 (Fig. 2D,E).

### Dynamic gene expression profiles in mice lung epithelial cells

**Figure 3:**
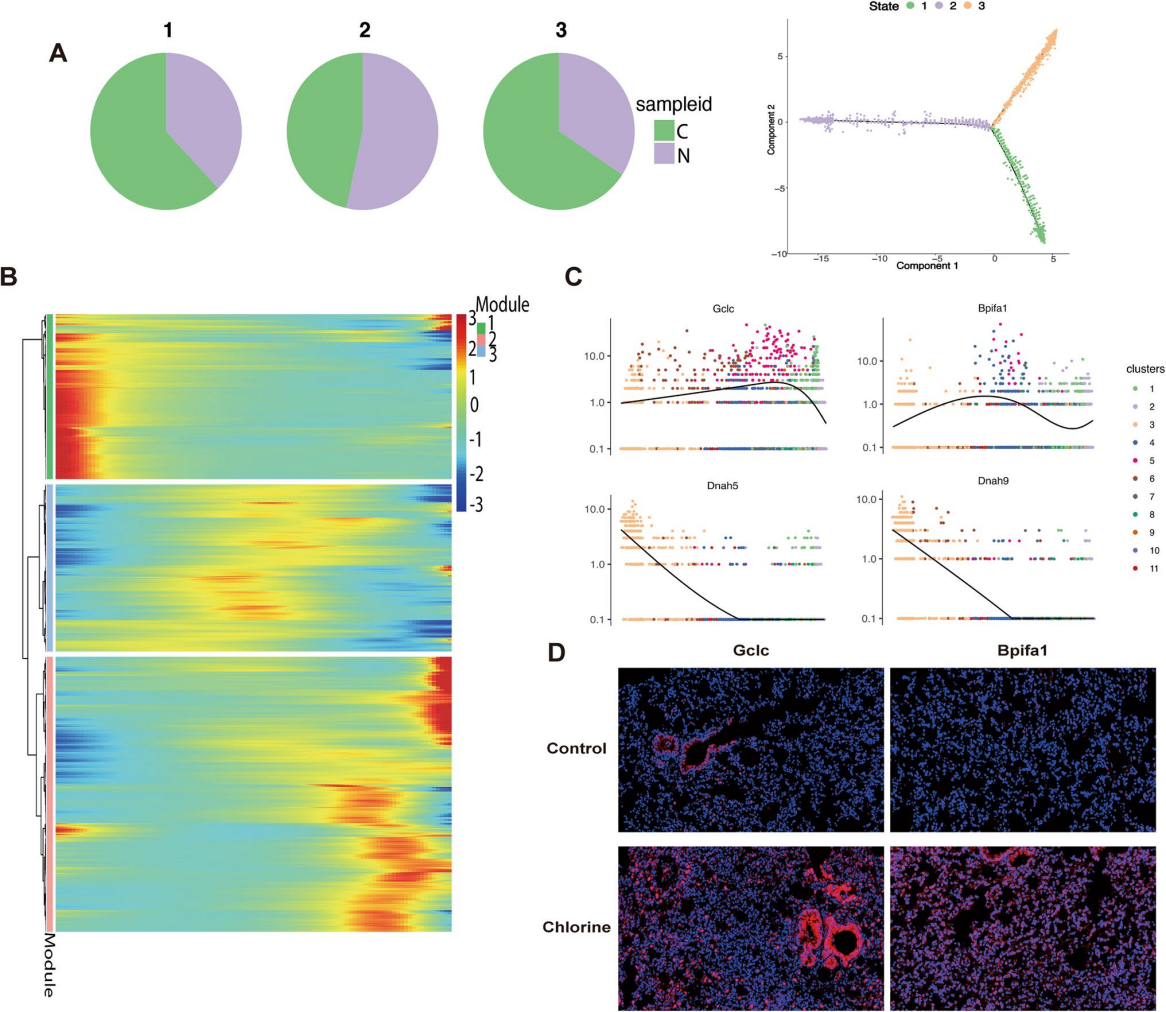
Pseudo-time analysis of epithelial cells. (**A**) Cell trajectories on pseudotime of lung epithelial cells. (**B**) Pseudotemporal dynamics of pseudotime-dependent genes in lung epithelial cells. Each row is normalized to its peak value along the pseudotime. (**C**) Pseudotime ordered single-cell expression trajectories for Gclc, Bpifa1, Dnah5 and Dnah9. (**D**) Immunofluorescence analysis of Gclc and Bpifa1. Scale bars 50 μm, n = 6, Gclc and Bpifa1(red), cell nuclear (blue), *N* control group, *C* Cl_2_ group.

To further elucidate the potential transition between normal and Cl_2_ injury epithelial cells, we conducted a pseudo-time analysis of epithelial cells. As shown in Fig. 3A, the state 2 with the highest proportion of normal epithelial cells was set as the root state in our study. Along this trajectory, the percentage of Cl_2_ damaged epithelial cells increased in the Cl_2_-exposed cells populations (Fig. 3A). Next, we also examined the activation of multiple key genes involved in lung epithelial cells differentiation. In exposure group, the expression of genes related to inhibition of ferroptosis and anti-inflammatory, such as Gclc and Bpifa1, showed a gradually increasing trend (Fig. 3B,C). The results of immunofluorescence staining also showed that the expression of Gclc and Bpifa1 increased after Cl_2_-exposure (Fig. 3D). Dnah5 and Dnah9 showed a decreasing trend (Fig. 3B,C).

### Pulmonary endothelial cells heterogeneity in the mice

**Figure 4:**
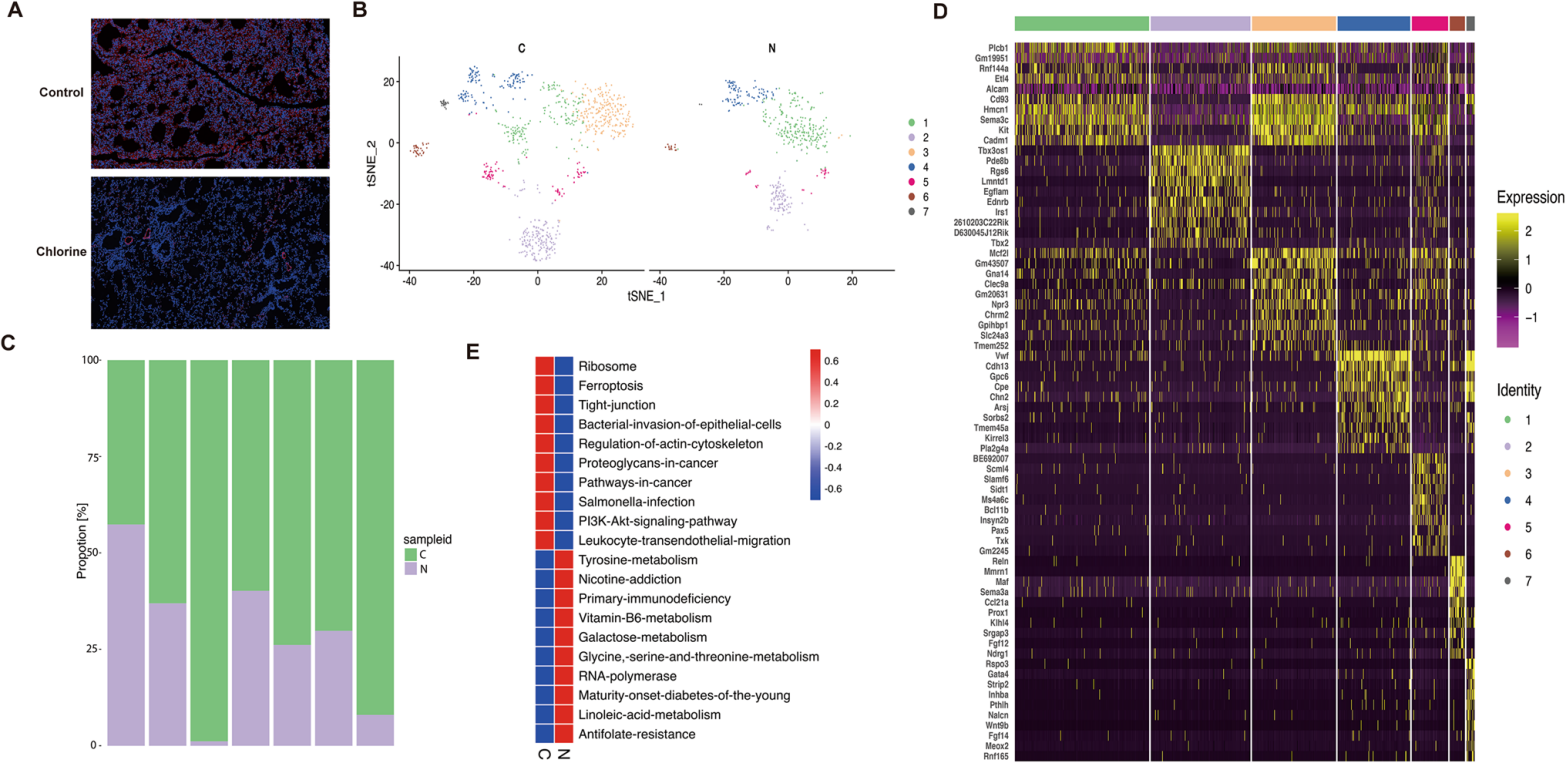
The single-cell transcriptomes of endothelial cells. (**A**) Immunofluorescence staining of endothelial cell Pecam1 gene in normal (n = 6)/Cl_2_-exposed (n = 6) lung tissues, scale bars 50 μm, Pecam1(red), cell nuclear (blue). (**B**) The t-SNE plot and overview of the endothelial cells. (**C**) Proportions of different cell types vary across in each subgroups of endothelial cells. Raw data of proportions of endothelial cells are shown in Supplementary information 4. (**D**) The heatmap shows the top 10 SDE genes in each subgroups of endothelial cells. (**E**) GSVA analysis indicated enrichment pathways of each subgroups of endothelial cells. *N* control group, *C* Cl_2_ group.

We performed immunofluorescence staining with endothelial cell marker gene Pecam1 in normal and Cl_2_-exposed mice lung tissues (Fig. 4A). The expression of Pecam1 was significantly decreased after injury. Combined with the data set identification results and the comprehensive analysis of the endothelial cells marker genes (Pecam1, CDH5, Vwf) identification results in the literature, cluster 4, 15, 18, 19, 21 may be endothelial cells. Next, we tried to describe the diversity of endothelial cells more completely, these endothelial cells were further divided into seven subclusters (endothelial cells cluster EC1 to EC7) through the SNN algorithm and t-SNE analysis (Fig. 4B). Interestingly, EC3 appeared after Cl_2_ exposure, and the position of each subcluster of endothelial cells in the exposed group also were changed to some extent compared with the control group (Fig. 4B). Compared with the control group, the subclusters of the lung endothelial cells in the Cl_2_ exposure group were significantly changed. The proportions of EC2, EC3, EC4, EC5, EC6, EC7 were increased, and only EC1 was decreased (Fig. 4C).

The unique gene characteristics of each endothelial cell subgroup and the top 10 SDE genes were described (Fig. 4D). Furthermore, GSVA analyses showed that some signaling pathways were differentially regulated by comparing control group with Cl_2_-exposure group (Fig. 4E). Compared with the endothelial cells in the control group, the signaling pathways such as ferroptosis, tight junction, regulation of actin cytoskeleton, PI3K/AKT, and leukocyte-endothelial-transmigration were highly enriched in the endothelial cells exposed to Cl_2_ (Fig. 4E). EC1 showed genes were associated with tight junctions: Alcam, Cadm1, Hmcn1 (Fig. 4D,E). The high expression genes Lmntd1 and Meox2 of EC2 and EC7 were related to the regulation of PI3K/AKT signaling pathway (Fig. 4D,E). Gpihbp1, Mmrn1 and Prox1 were highly expressed in EC3 and EC6 respectively, and they were all closely related to the regulation of actin cytoskeleton in EC4 overexpression genes Cdh13 and Sorbs (Fig. 4D,E). The highly expressed genes Bcl11b and Slamf6 in EC5 were all closely related to the regulation of actin cytoskeleton. Cdh13 and Sorbs in EC4 overexpression genes showed a correlation with the regulation of immune inflammation (Fig. 4D,E).

### Crosstalk between epithelial–endothelial cells

To systematically study the interaction between epithelial and endothelial cells in the mice lung tissues with Cl_2_-induced ALI, the study used siscRNA-seq to explore the gene expression data of cell subgroups with the help of a ligand–receptor database. Using cellphoneDB, we can obtain information on intracellular ligands and receptors, and obtain intercellular signaling communication relationships to elucidate the complexity, diversity and dynamics of communication between epithelial and endothelial cells during Cl_2_-induced ALI.

**Figure 5:**
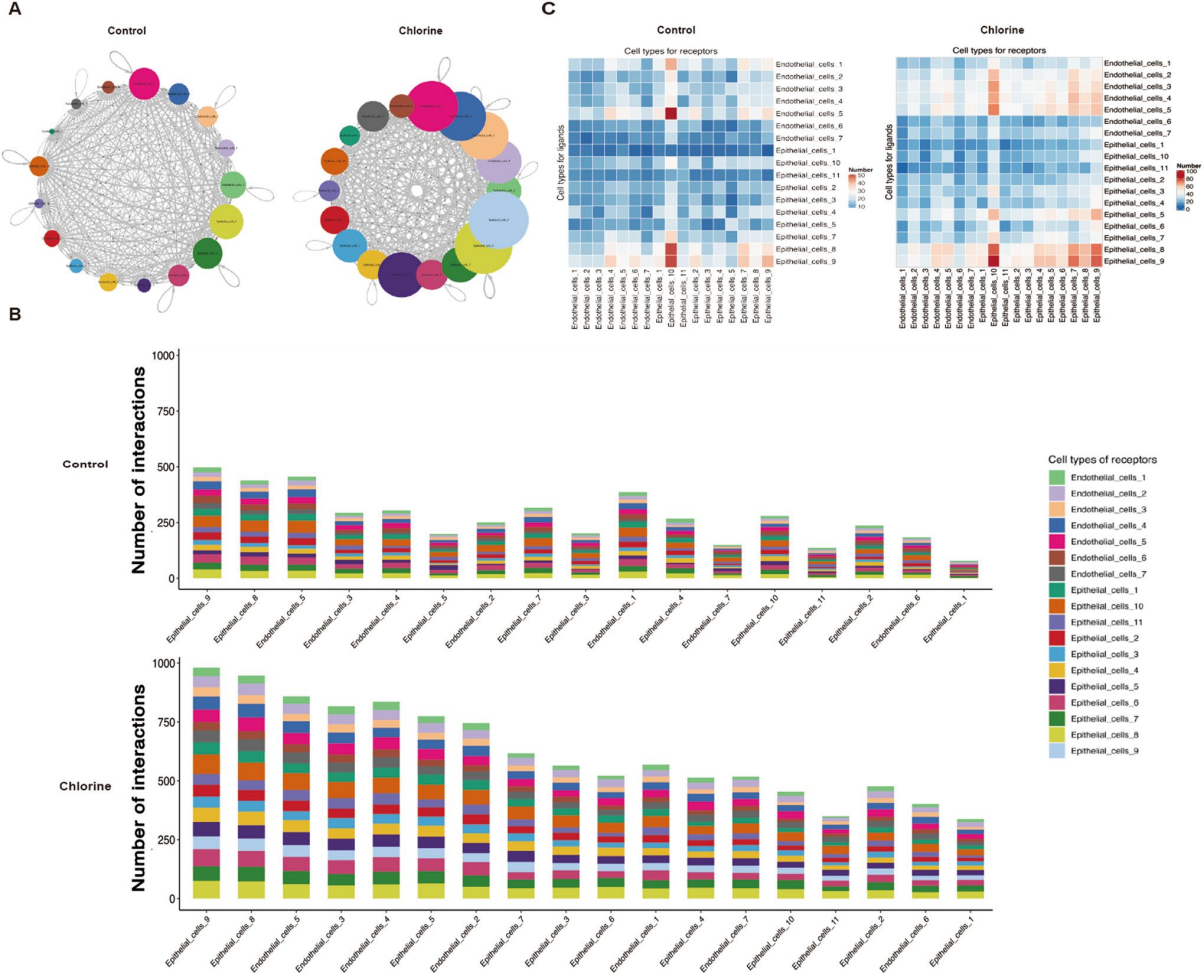
Intercellular epithelial–endothelial cells communication in mice lung tissues. (**A**) Capacity for intercellular communication among epithelial and endothelial cells. The nodes in the figure represent different cell types, the arrows represent the interaction signals from ligand cells to receptor cells, and the numbers on the arrows represent the number of significant ligand–receptor interaction pairs detected between different cell types. (**B**) The number of ligand receptors for epithelial–endothelial cells1interactions was shown by stacked bar graphs. (**C**) Heatmap shown the total number of interactions between cell types. The redder the color, the higher the number of interacting ligand–receptor. *N* control group, *C* Cl_2_ group.

**Figure 6:**
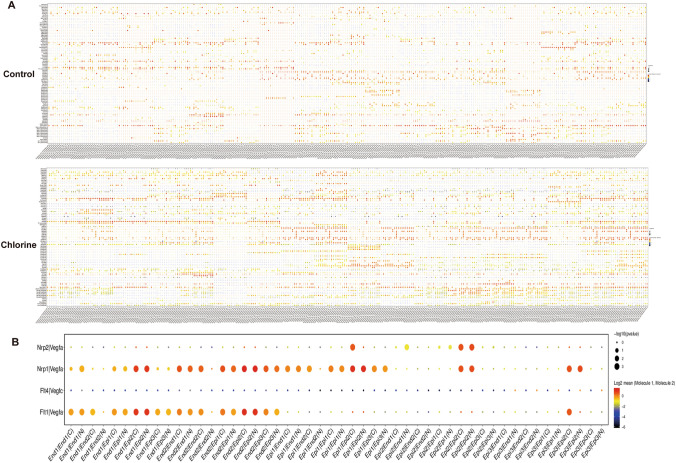
Intercellular epithelial–endothelial cells communication regulation based on ligand–receptor analysis. (**A**) Overview of selected ligand receptor interactions of epithelial and endothelial cells. (**B**) Nrp1-Vegfa, Nrp2-Vegfa, Flt1-Vegfa and Flt4-Vegfa of selected ligand receptor interactions of epithelial and endothelial cells. *N* control group, *C* Cl_2_ group.

The study revealed a complex network of cell–cell interactions between epithelial and endothelial cells (Fig. 5A). Compared with the control group, the number of receptor ligand interaction in each cluster of epithelial–endothelial cells was significantly increased in the exposed group (Fig. 5B). We found that at homeostasis, the highest number of putative epithelial–endothelial cells interactions was predicted to occur between ligand-producing cluster 10 epithelial cells and EC5 endothelial cells (Fig. 5C). After Cl_2_ injury, the data indicated that signaling interactions between cluster 10 epithelial cells and EC5 endothelial cells are predicted to remain high (Fig. 5C). The EC2, EC3 and EC4 endothelial cells subtypes were also predicted to be involved in signaling interactions with cluster 10 epithelial cells following Cl_2_ injury (Fig. 5C). Specifically, by further analysis the distinct receptor–ligand interactions were identified in the two groups (Fig. 6A). Vascular endothelial-derived growth factor (Vegf) acts as a protective factor in normal lung. Moreover, we also found that CD74 and cell adhesion molecule 1 (CADM1) were relatively highly expressed in the Cl_2_ exposure group (Fig. 6A). In addtion, in the Cl_2_ exposure group, a variety of Vegfa-related receptor–ligands were identified in interaction among Nrp1-Vegfa, Nrp2-Vegfa, Flt1-Vegfa and Flt4-Vegfa (Fig. 6B).

### Ferroptosis can drive Cl_2_-induced ALI

We found that ferroptosis was activated in the KEGG analysis of total cells. At the same time, in the GSVA analysis of epithelial and endothelial cells, the results were consistent with KEGG analysis (Figs. 1F, 2E, 4E).

**Figure 7:**
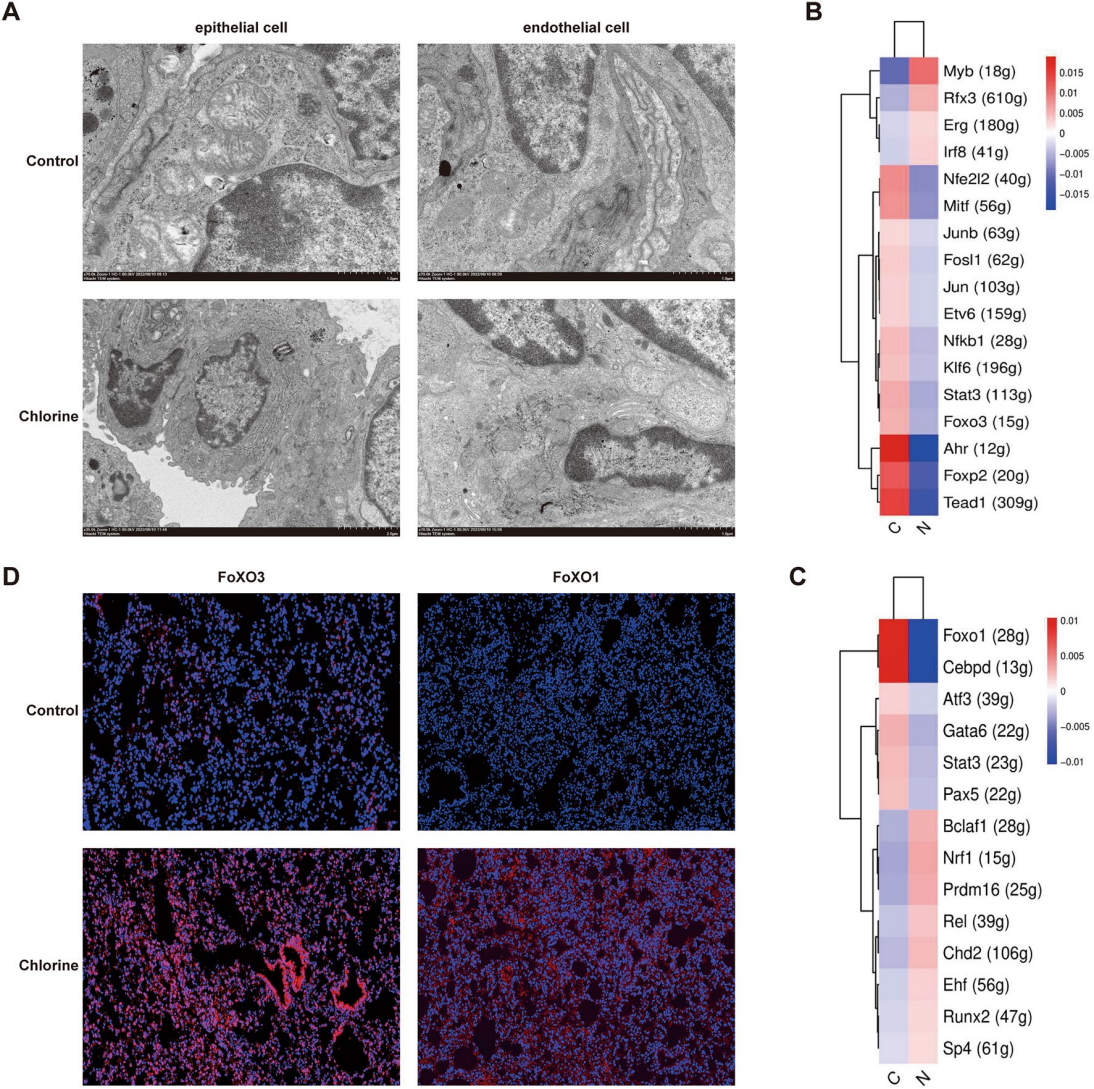
The expression of mice ferroptosis after Cl_2_ exposure. (**A**) The transmission electron microscopy observation of epithelial and endothelial cells, scale bars 70 μm, n = 6. (**B**,**C**) SCENIC analyses of epithelial and endothelial cells in control and Cl_2_-exposed groups. (**D**) Immunofluorescence staining of FoXO1 and FoXO3 genes, scale bars 50 μm, n = 6, FoXO1 and FoXO3 (red), cell nuclear (blue), *N* control group, *C* Cl_2_ group.

In mice lung tissues, through the TEM we found mitochondrial cristae of epithelial and endothelial cells fractured, and the density of cell membrane increasing in the Cl_2_-exposure group (Fig. 7A), which indicated that ferroptosis occurred after Cl_2_-exposed.

SCENIC analysis was used to infer the regulatory activity of epithelial and endothelial cells (Fig. 7B,C). The genes FoxO3 and FoxO1 were respectively upregulated in the epithelial and endothelial cells of the Cl_2_ exposure group (Fig. 7B,C). Next, we performed immunofluorescent staining to provide evidence that FoXO1 and FoXO3 were increased in mice lung tissues after Cl_2_ exposure (Fig. 7D). These indicated the existence of the above speculations.

### PTX can inhibit ferroptosis

**Figure 8:**
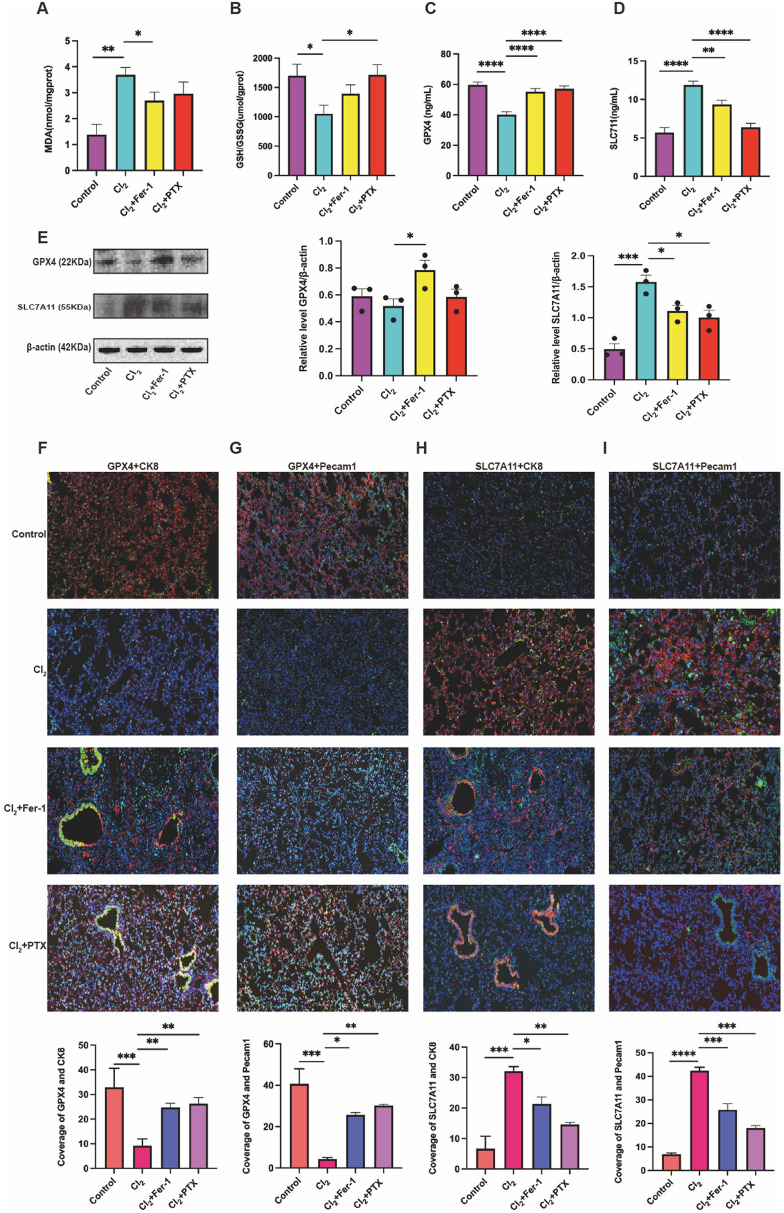
PTX inhibit Cl_2_-induced ferroptosis. (**A**,**B**) The expression levels of MDA, GSH/GSSG in mice serum treated with Cl_2_, n = 8. (**C**,**D**) Protein levels of GPX4 and SLC7A11 in mice lung and serum treated with Cl_2_, n = 8. (**E**) GPX4 and SLC7A11 protein levels were determined by western blot analysis, and standardized to the expression of β-actin. Original scans of whole blots are shown in Supplementary information 5. (**F**,**G**) Immunofluorescence images were used to detect the expression of GPX4 in epithelial and endothelial cells, scale bars 50 μm, n = 6. (**H**,**I**) Immunofluorescence images were used to detect the expression of SLC7A11 in epithelial and endothelial cells, scale bars 50 μm, n = 6. Epithelial cell: CK8 (green), endothelial cells: Pecam1 (green), GPX4 (red), SLC7A11 (red), cell nuclear (blue). The data was shown as mean ± SEM, *P < 0.05, **p < 0.01, *p* < 0.001, ****p < 0.0001, n = 8.

To test whether ferroptosis plays an important role in Cl_2_-induced ALI and the therapeutic action of PTX, Fer-1 was used as a positive control. As shown in Fig. 8A,B, Cl_2_ exposure increased the level of MDA and reduced the activity of GSH/GSSG (Fig. 8A,B), while PTX and Fer-1 interventions could inhibit the expression of MDA and increase the ratio of GSH/GSSG (Fig. 8A,B).

Next, we also evaluated the GPX4 and SLC7A11 expression in mice lung tissues and serum (Fig. 8C–E). The level of GPX4 was decreased in the Cl_2_ group compared with the control group. On the contrary, SLC7A11 was elevated in the exposure group compared with the control group, which may be related to its increased irritability (Fig. 8C–E). PTX could increase the expression of GPX4 and decrease the abnormal high expression of SLC7A11 in lung tissues and serum, especially SLC7A11 (p < 0.05), compared with Cl_2_ group (Fig. 8C–E). The expression level of GPX4 in Cl_2_+PTX group did not improve significantly than Cl_2_ group, but the GPX4 expression level did increase compared with Cl_2_ group (Fig. 8C).

The expression level of GPX4 and SLC7A11 was detected by immunofluorescence staining in epithelial and endothelial cells (Fig. 8F–I). We found that the expression level of GPX4 and SLC7A11 were the same change in trend as the above results (Fig. 8C–I). PTX and Fer-1 significantly increase the expression of GPX4 and improve the abnormal high expression of SLC7A11 in epithelial and endothelial cells, though the interventional effect of PTX slightly better than Fer-1 (Fig. 8F–I). These data indicate that PTX could improve Cl_2_-induced ALI by inhibiting ferroptosis.

## Discussion

Cl_2_ is a toxic respiratory irritant and is considered as a chemical threat agent. In ALI caused by Cl_2_, the function of blood air barrier is first affected. Epithelial and endothelial cells are the main components. Their early molecular events remain unclear. This study showed that the signaling pathway which regulates the cytoskeleton were not only highly expressed, but also the top10 highly expressed genes such as Tmem132d, Dlg4, Cnksr2 and Fgd5 were related to it. It is known that when lung epithelial cells are exposed to injurious stimuli, they dynamically regulate the arrangement and expression of cytoskeletal and tight junction proteins thereby causing changes in intercellular adhesion and thus affecting intracellular homeostasis^18^, which is consistent with the findings of this study. This suggested that repairing cytoskeleton of epithelial cells may be critical for Cl_2_-induced epithelial cells injury.

In pseudo-time analysis of epithelial cells, we found that Bpifa1 secreted by airway epithelial cells, showed a gradually increasing trend. Previous study has shown that Bpifa1 has immunomodulatory function for airway inflammatory diseases^19^. Hence, inflammation plays an important negative role in the damage of epithelial cells. 80% of healthy human airway epithelial cells are ciliated cells, and abnormal ciliary motor function will lead to decreased airway defense function^20^. Dnah5 and Dnah9 are related to ciliary dyskinesia^20,21^, which showed a decreased trend. The finding suggested that the barrier function of lung epithelial cells might be disrupted after Cl_2_ exposure, and lung epithelial cilia were gradually repaired over time. The results may be closely correlated with the gradual repair of injured lung tissue after Cl_2_ exposure.

In this study, we identified top 10 genes that were highly expressed in each endothelial cell subpopulation. In addition, some signaling pathways that were highly expressed by GSVA analysis were compared to the control group. Previously, it was shown that ALI leads to increased endothelial permeability in the pulmonary vasculature, which is closely associated with reduced expression of tight junction proteins^22^. This study showed that signaling pathways and genes were associated with tight junction proteins highly expressed in endothelial cells. We also found that tight junction proteins signaling pathways and genes were highly expressed not only in endothelial cells but also in the interaction of epithelial and endothelial cell receptor–ligand, which is consistent with the results reported by Liu et al.^22^.

The critical role of the barrier between pulmonary epithelial or endothelial cells have been demonstrated, but the role of cell communication for epithelial–endothelial cells in Cl_2_-induced ALI was not reported^23–25^. Herein, by dissecting the epithelial–endothelial cell communication several receptor–ligands were identified, which should be critical for the development of Cl_2_-induced ALI. We found that Vegfa-related receptor–ligands, CD74 and CADM1 were highly expressed in the Cl_2_-exposure group. It has been previously reported that CD74 is involved in an inflammatory response to ALI, which leads to disruption of pulmonary vascular epithelial and endothelial cells barrier function^26,27^. These suggest that epithelial and endothelial cells simultaneously play important roles in Cl_2_-induced ALI.

Ferroptosis was first reported by Dr Brent R. Stockwell in 2012^28^. It is a newly recognized type of cell death that differs from traditional necrosis, apoptosis or autophagic cell death^29^. The inactivation of cell antioxidant system is a major reason for ferroptosis, which mainly results from decrease of Xc^-^ activity system, GPX4, and increase lipid reactive oxygen species (ROS)^30^. Glutathione peroxidases (GPXs) gene belongs to the antioxidant family, which is the main selenoprotein in human body. Compared with other GPXs members, GPX4 directly reduces lipid hydrogen peroxide into non-toxic lipid alcohols in the membrane and acts as the main regulator in the process of ferroptosis^31^. In addition, SLC7A11, encoding cystine/glutamate xCT transporter, is also a key gene involved in regulating ferroptosis^32^. By applying KEGG and GSVA analyses, we found that ferroptosis was highly expressed in both epithelial and endothelial cells after Cl_2_ exposure. TEM results also verified this discovery. Pseudo-time analysis of epithelial cells also showed that the expression of Gclc, which helps to ROS ^33,34^ increased gradually during the progression of the disease and could effectively inhibit the development of ferroptosis. The specific mechanism of Gclc in ferroptosis needs further study. We found two genes which related to antioxidant damage by SCENIC analysis—FoXO3 and FoxO1. We also confirmed by immunofluorescence staining that the expression of FoXO1 and FoxO3 were indeed elevated. It was previously reported that in vitro and in vivo experiments knockout of the three genes FoxO1, FoxO3a and FoxO4 increased the production of ROS^35,36^. Hence, we speculate that FoxO3 and FoxO1 may be closely related to the progression of ferroptosis because of the role of FoxOs’ in fighting against oxidative stress. But it needs to be proved.

PTX, as a vasoactive agent, has been used for clinical observation in COVID-19, but lacks in its laboratory data^9,37^. Our research group observed the interventional effect of PTX in animals in the early stage, and found that PTX could inhibit the inflammatory reaction caused by phosgene^38^ and improve the oxidative damage caused by Cl_2_^39^. They reported that PTX could reduce oxidative damage in the lung and protect ALI caused by Cl_2_ exposure by affecting the number and function of mitochondria^39,40^. Based on previous study, the interventional effect of PTX on ferroptosis was observed in this study. This study found that PTX could significantly decrease the accumulation of MDA while increase the ratio of GSH/GSSG. Importantly, PTX improve the expression of GPX4 and decrease the abnormal high expression of SLC7A11 in the lung tissue, which are characteristic indicators of ferroptosis. In order to further observe the effect of PTX on ferroptosis, we detected the expression level of GPX4 and SLC7A11 in lung tissue by WB andimmunofluorescence staining. In particular, they were found to mainly expressed in epithelial and endothelial cells. Hence, it is demonstrated that PTX could against ferroptosis in epithelial and endothelial cells. In addition, based on the safety of PTX (FDA approved for the symptomatic treatment of claudication^8^) in clinical use, as well as its broad pharmacological effects such as anti-inflammatory, antioxidant and microcirculation improvement, PTX offer a glimpse of considerations for future use as a potential drug to Cl_2_-induced ALI treatment.

In a nutshell, the study reveals heterogeneity of Cl_2_-induced mice ALI by scRNA-seq. We conducted a series of in-depth analyses of epithelial and endothelial cells in normal and Cl_2_-exposed mice. Several novel genes, biological functions and signaling pathways such as regulation of actin cytoskeleton, junction proteins, PI3K/AKT and so on, which facilitated a deeper understanding of the functions and roles of epithelial and endothelial cells in the injury progresses. Especially, we found ferroptosis and its related genes, which provided potential new targets and ideas for future treatment of Cl_2_-induced ALI. The most meaningful significance for the study is that we verified that PTX could improve of Cl_2_-induced ferroptosis.

## Supplementary Information

Supplementary Information 1.

Supplementary Information 2.

Supplementary Information 3.

Supplementary Information 4.

Supplementary Information 5.

## Data Availability

The datasets used and analyzed in this study are available from the corresponding author on reasonable request. Moreover, the KEGG database, GSVA and scFunctions packages of this study are available at www.kegg.jp/kegg/kegg1.html, http://www.bioconductor.org/packages/release/bioc/html/GSVA.html and https://github.com/FloWuenne/scFunctions/. The remaining data are available within the article and its supplementary information.
