# Supplementary material for: Single-cell transcriptomes reveal heterogeneity of chlorine-induced mice acute lung injury and the inhibitory effect of pentoxifylline on ferroptosis
Source: Sci Rep. 2023 Apr 26;13:6833. doi: 10.1038/s41598-023-32093-7 (PMC10131515; doi:10.1038/s41598-023-32093-7)
Supplement: Supplementary file 1 — Supplementary Information 1. [file 41598_2023_32093_MOESM1_ESM.pdf]

Quality control of gene quantification in control group samples

10x GENOMICS Cell Ranger • count

N - HT2021-17640-1_小鼠-肺组织

Alerts

The analysis detected 1 error and 1 warning.

Alert	Value	Detail
Low Fraction Reads in Cells	34.6%	Ideal > 70%. Application performance may be affected. Many of the reads were not assigned to cell-associated barcodes. This could be caused by high levels of ambient RNA or by a significant population of cells with a low RNA content, which the algorithm did not call as cells. The latter case can be addressed by inspecting the data to determine the appropriate cell count and using --force-cells.
High Fraction of Reads Mapped Antisense to Genes	10.3%	Ideal < 10%. This can indicate use of an unsupported chemistry type (e.g. using Single Cell V(D)J for gene counting). Application performance is likely to be affected.

Summary | Analysis

6,226
Estimated Number of Cells

91,350
Mean Reads per Cell

735
Median Genes per Cell

Sequencing

Number of Reads	568,746,316
Number of Short Reads Skipped	0
Valid Barcodes	95.6%
Valid UMIs	99.6%
Sequencing Saturation	92.0%
Q30 Bases in Barcode	96.3%
Q30 Bases in RNA Read	93.3%
Q30 Bases in UMI	96.0%

Mapping

Reads Mapped to Genome	91.2%
Reads Mapped Confidently to Genome	88.3%
Reads Mapped Confidently to Intergenic Regions	10.4%
Reads Mapped Confidently to Intronic Regions	40.5%
Reads Mapped Confidently to Exonic Regions	37.4%
Reads Mapped Confidently to Transcriptome	67.3%
Reads Mapped Antisense to Gene	10.3%

Cells

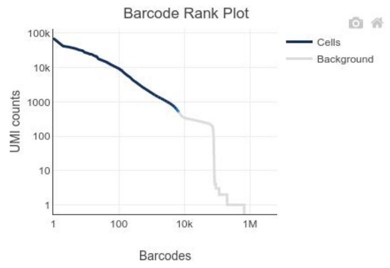

Estimated Number of Cells	6,226
Fraction Reads in Cells	34.6%
Mean Reads per Cell	91,350
Median Genes per Cell	735
Total Genes Detected	23,383
Median UMI Counts per Cell	985

Sample

Sample ID	N
Sample Description	HT2021-17640-1_小鼠-肺组织
Chemistry	Single Cell 3' v3
Include introns	True
Reference Path	…nger-refdata/refdata-gex-mm10-2020-A
Transcriptome	mm10-2020-A
Pipeline Version	cellranger-5.0.0

Quality control of gene quantification in Cl_2 group samples

10x GENOMICS Cell Ranger • count

C - HT2021-17640-1_小鼠-肺组织

Alerts

The analysis detected ⚠ 2 warnings.

	Alert	Value	Detail
⚠	High Fraction of Reads Mapped Antisense to Genes	25.0%	Ideal < 10%. This can indicate use of an unsupported chemistry type (e.g. using Single Cell V(D)J for gene counting). Application performance is likely to be affected.
⚠	Low Fraction Reads in Cells	51.6%	Ideal > 70%. Application performance may be affected. Many of the reads were not assigned to cell-associated barcodes. This could be caused by high levels of ambient RNA or by a significant population of cells with a low RNA content, which the algorithm did not call as cells. The latter case can be addressed by inspecting the data to determine the appropriate cell count and using --force-cells.

Summary | Analysis

9,659
Estimated Number of Cells

67,645
Mean Reads per Cell

1,324
Median Genes per Cell

Sequencing

Number of Reads	653,381,595
Number of Short Reads Skipped	0
Valid Barcodes	94.3%
Valid UMIs	99.7%
Sequencing Saturation	79.4%
Q30 Bases in Barcode	96.4%
Q30 Bases in RNA Read	91.9%
Q30 Bases in UMI	96.1%

Mapping

Reads Mapped to Genome	88.3%
Reads Mapped Confidently to Genome	85.5%
Reads Mapped Confidently to Intergenic Regions	8.6%
Reads Mapped Confidently to Intronic Regions	41.2%
Reads Mapped Confidently to Exonic Regions	35.7%
Reads Mapped Confidently to Transcriptome	51.7%
Reads Mapped Antisense to Gene	25.0%

Cells

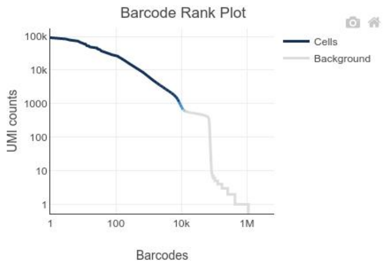

Estimated Number of Cells	9,659
Fraction Reads in Cells	51.6%
Mean Reads per Cell	67,645
Median Genes per Cell	1,324
Total Genes Detected	26,028
Median UMI Counts per Cell	2,104

Sample

Sample ID	C
Sample Description	HT2021-17640-1_小鼠-肺组织
Chemistry	Single Cell 3' v3
Include introns	True
Reference Path	…nger-refdata/refdata-gex-mm10-2020-A
Transcriptome	mm10-2020-A
Pipeline Version	cellranger-5.0.0
