# Supplementary figures and images for: Single-cell transcriptomes reveal heterogeneity of chlorine-induced mice acute lung injury and the inhibitory effect of pentoxifylline on ferroptosis
Source: Sci Rep. 2023 Apr 26;13:6833. doi: 10.1038/s41598-023-32093-7 (PMC10131515; doi:10.1038/s41598-023-32093-7)

Uncropped immunoblots in Figure. 8E

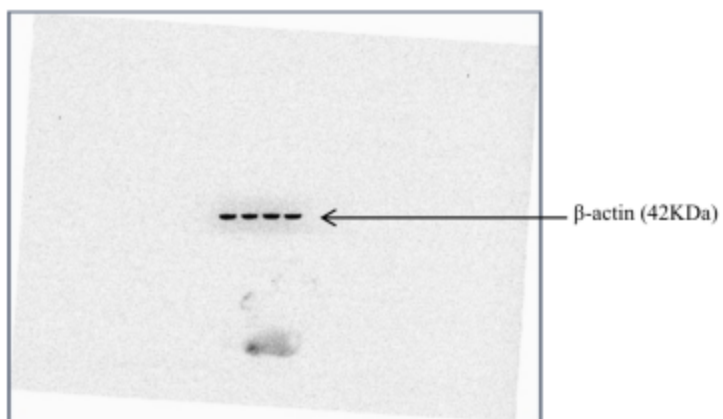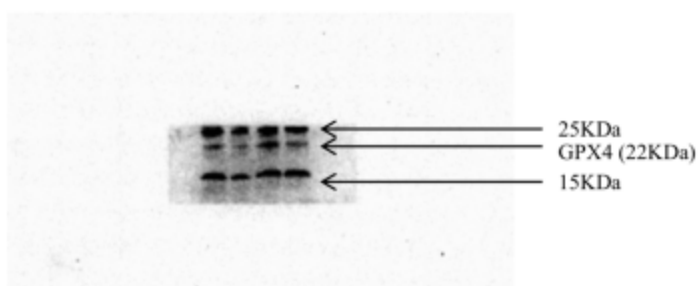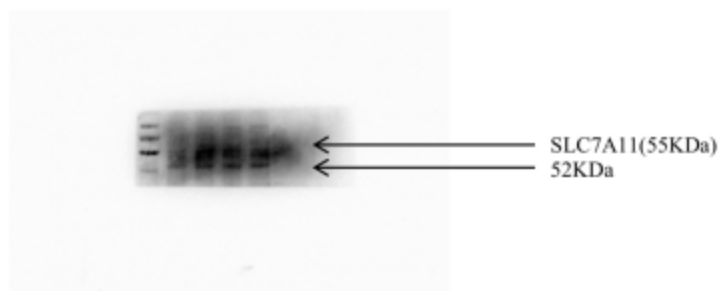

Supplement: Supplementary file 5 — Supplementary Information 5. [file 41598_2023_32093_MOESM5_ESM.pdf]
